# 

**Published:** 1890-02-22

**Authors:** 


SATURDAY, FEBRUARY 22nd, 1890.

**A Rare Event** - 319
**Words of Consolation.**—Perfection - 320
**The History and Capabilities of Herbal Simples.**—V. The Common Arum, Asparagus, and Balm - 321
**The Moral Basis of Private Property.**—I. - 322
**The Doctor's Pulpit.**—Elixers and Inoculations - 323
**The Hospital for the Insane** - 324
**Everybody's Page** - 325
**Notes and News** - 326
**Annotations.**—Influenza—London Paupers to the Country—Benzon and His Friends - 328
**Turning Over New Leaves** - 333
**Editor's Letter Box** - 333
**The Practitioner's Outlook and Retrospect:**
MEDICINE—Diphtheretic Paralysis—Hydrophobia—Puerperal Fevers - 329
SURGERY—The Surgical Aspect of Hepatic Abscess—The Temporary Ligature - 330
THERAPEUTICS—Cocaine—Sulphonal - 331
CLIMATOLOGY—Cornwall as a Winter Resort - 331
DIETETICS—Milk for Infants - 332
NEW DRUGS, APPLIANCES, AND THINGS MEDICAL—Van Houten's Cocoa—Tucker's Eucalyptus Preparations - 332
**Passing Topics.**—Ought Members of the Active Medical Staff to Sit upon Hospital Boards? - 334
**Scraps and Gleanings** - 334

### EXTRA SUPPLEMENT—THE NURSING MIRROR.

**En Passant.**—Infirmary Nurses and the Pension Fund—Short Items—District Nursing at Glasbury—Tired American Nurses—Nurses for the Poor—Thousands of Pounds for the National Pension Fund - lxxxv
**Nursing Lectures.** By PERCY LEWIS, M.D. III. Symptoms in Chest Diseases - lxxxvi
**Death in Our Ranks** - lxxxviii
**The Nurses' Bookshelf.**—District Nursing—Nursing for Sixpence—Nursing Questions and Answers - lxxxvii
**Everybody's Opinion.**—Meum and Tuum — A First Operation Case - lxxxvii
**Notes and Queries** - lxxxviii
**Appointments and Vacancies** - lxxxviii
**Amusements and Relaxation** - lxxxviii

